# Suppression of APOBEC3-mediated restriction of HIV-1 by Vif

**DOI:** 10.3389/fmicb.2014.00450

**Published:** 2014-08-26

**Authors:** Yuqing Feng, Tayyba T. Baig, Robin P. Love, Linda Chelico

**Affiliations:** Department of Microbiology and Immunology, College of Medicine, University of SaskatchewanSaskatoon, SK, Canada

**Keywords:** HIV, mutagenesis, deaminase, Vif, APOBEC3, restriction factor, ubiquitination

## Abstract

The APOBEC3 restriction factors are a family of deoxycytidine deaminases that are able to suppress replication of viruses with a single-stranded DNA intermediate by inducing mutagenesis and functional inactivation of the virus. Of the seven human APOBEC3 enzymes, only APOBEC3-D, -F, -G, and -H appear relevant to restriction of HIV-1 in CD4+ T cells and will be the focus of this review. The restriction of HIV-1 occurs most potently in the absence of HIV-1 Vif that induces polyubiquitination and degradation of APOBEC3 enzymes through the proteasome pathway. To restrict HIV-1, APOBEC3 enzymes must be encapsidated into budding virions. Upon infection of the target cell during reverse transcription of the HIV-1 RNA into (-)DNA, APOBEC3 enzymes deaminate cytosines to form uracils in single-stranded (-)DNA regions. Upon replication of the (-)DNA to (+)DNA, the HIV-1 reverse transcriptase incorporates adenines opposite to the uracils thereby inducing C/G to T/A mutations that can functionally inactivate HIV-1. APOBEC3G is the most studied APOBEC3 enzyme and it is known that Vif attempts to thwart APOBEC3 function not only by inducing its proteasomal degradation but also by several degradation-independent mechanisms, such as inhibiting APOBEC3G virion encapsidation, mRNA translation, and for those APOBEC3G molecules that still become virion encapsidated, Vif can inhibit APOBEC3G mutagenic activity. Although most Vif variants can induce efficient degradation of APOBEC3-D, -F, and -G, there appears to be differential sensitivity to Vif-mediated degradation for APOBEC3H. This review examines APOBEC3-mediated HIV restriction mechanisms, how Vif acts as a substrate receptor for a Cullin5 ubiquitin ligase complex to induce degradation of APOBEC3s, and the determinants and functional consequences of the APOBEC3 and Vif interaction from a biological and biochemical perspective.

## OVERVIEW

**FIGURE 1:**
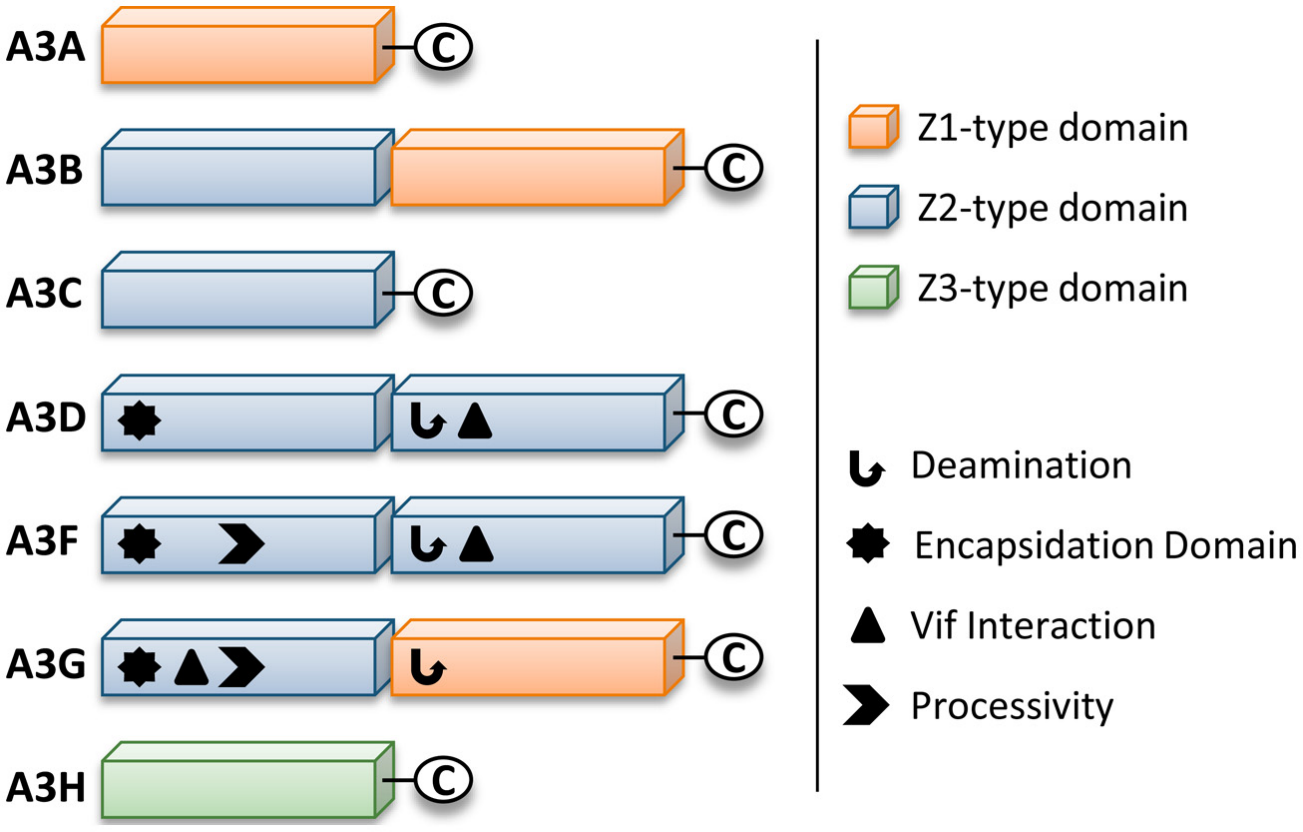
**Zinc (Z) coordinating-type domains of human A3 enzymes.** A3 enzymes coordinate zinc through the motif H-X-E-X_23-28_-P-C-X_2-4_-C. The glutamate activates a water molecule to enable zinc-hydroxide-mediated nucleophilic attack to complete the deamination reaction. Deamination activity has been demonstrated for all A3 enzymes. For the enzymes with two Z-type domains that restrict HIV in CD4+ T cells (A3D, A3F, and A3G), a legend depicts known biochemical functions of each Z-type domain. A common feature of A3 enzymes with two Z-type domains is the segregation of functions in the N-terminal domain (NTD) and C-terminal domain (CTD). The NTD is responsible for encapsidation and the CTD is responsible for deamination activity. Both domains can bind nucleic acids. The binding site of Vif is in the NTD for A3G and in the CTD for A3D and A3F. The determinants for enzyme processivity have only been studied for A3G and A3F. A3G and A3F processivity is imparted by the NTD.

Retrotransposons and endogenous retroviruses have been genomic parasites in organisms throughout evolution and have contributed to both species evolution and disease (Hancks and Kazazian, 2012). The APOBEC (*Apolipoprotein B* mRNA-editing *e*nzyme-*c*atalytic polypeptide) family of enzymes present in their earliest form in bony fish acted as a defense to retroelements (MacDuff et al., 2009). Due to expansion of retroelements through evolution, there was a corresponding expansion in the APOBEC family (Jern and Coffin, 2008; LaRue et al., 2008). The most recent expansion in placental mammals formed the APOBEC-like *3* (APOBEC3) family in response to ancient pathogenic retroviruses (LaRue et al., 2008; Munk et al., 2012). Humans contain seven APOBEC3 (A3) enzymes (A3A, A3B, A3C, A3D, A3F, A3G, and A3H, Jarmuz et al., 2002; LaRue et al., 2009). The A3 enzymes act as host restriction factors to inhibit retroelement replication through either RNA binding ability or activity as single-stranded (ss) DNA cytosine deaminases that catalyze the formation of promutagenic uracils (Esnault et al., 2005; Bogerd et al., 2006; Chiu et al., 2006; Jonsson et al., 2006; Armitage et al., 2008; OhAinle et al., 2008; Khatua et al., 2010; Duggal et al., 2011; Koyama et al., 2013). Currently, A3 enzymes are primarily studied for their ability to restrict the replication of retroviruses (such as HIV-1, Sheehy et al., 2002; Harris et al., 2003; Mangeat et al., 2003; Zhang et al., 2003; Liddament et al., 2004; Wiegand et al., 2004; Zheng et al., 2004; Dang et al., 2006, 2008; OhAinle et al., 2008; Richardson et al., 2014) and other viruses with an ssDNA intermediate (such as Hepatitis B Virus, Kock and Blum, 2008; Lucifora et al., 2014). Restriction of the replication of these present day viruses occurs primarily through the deoxycytidine deamination activity of A3 enzymes which results in hypermutated and inactivated viral genomes. The gene duplications that resulted in the human A3 repertoire formed two general groups of deaminases with different Zinc (Z) coordinating domains: A3A, A3C, and A3H are enzymes with a single Z-domain and A3B, A3G, A3D, and A3F enzymes with two Z-domains (LaRue et al., 2008, **Figure 1**). For A3 enzymes with two Z-domains, only one domain is catalytically active, except for A3B, which may have two catalytically active domains (Hache et al., 2005; Navarro et al., 2005; Bogerd et al., 2007; Bonvin and Greeve, 2007, **Figure 1**).

**FIGURE 2:**
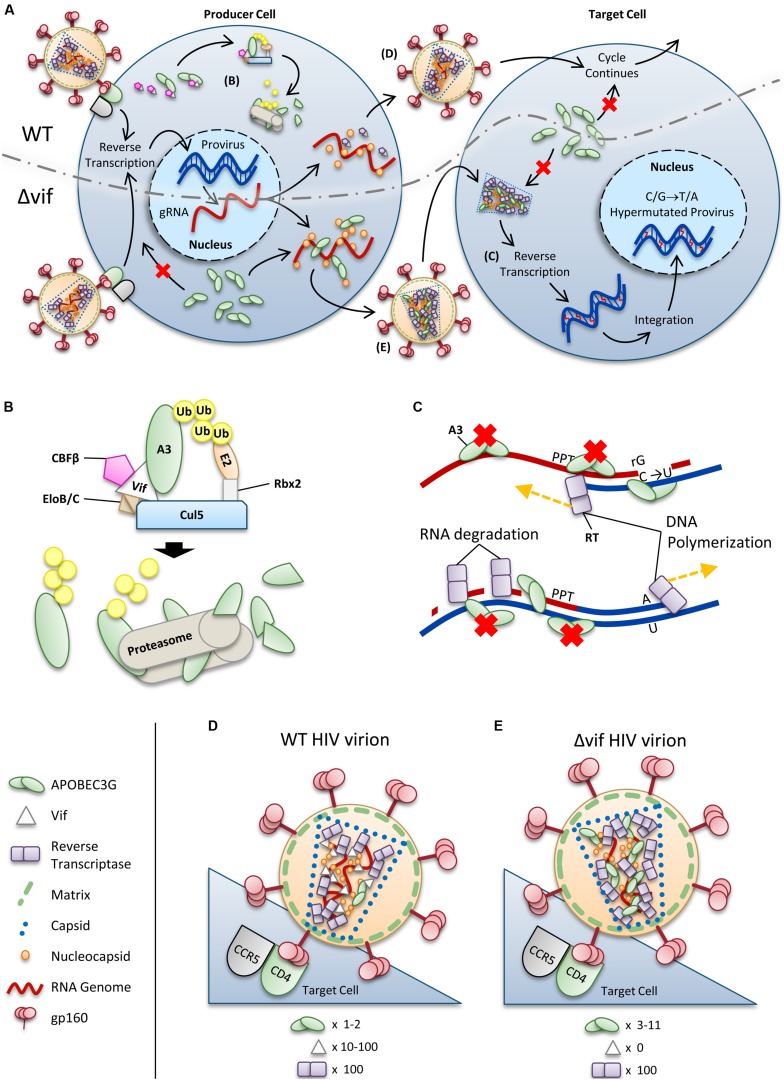
**Overview of HIV restriction by A3 enzymes. (A)** Sketch depicting lifecycles of wild-type (WT) and ΔVif HIV (ΔVif). Each virion enters a cell that expresses A3 enzymes. In the WT virus, Vif is expressed in the cell and recruits host cell CBFβ for stability and CRL5 E3 ubiquitin ligase complex composed of Elongin B/C (EloB/C), Cullin5 (Cul5) and Rbx2 (**B**). In this complex, Vif acts as the substrate receptor to induce degradation of A3 enzymes. As a result, assembling virions do not encapsidate high levels of A3 enzymes and upon infection of a target cell the HIV lifecycle continues. The ΔVif HIV encapsidates A3 enzymes through an RNA and Gag interaction. In the target cell the A3 enzymes within the capsid of HIV can deaminate cytosines to uracils in nascent single-stranded (-)DNA during reverse transcription (**C**). These uracils induce G→A transition mutations upon synthesis of (+)DNA (**C**). The resulting hypermutated virus can be integrated into the host genome but is functionally inactivated. A3 enzymes in the target cell cannot enter the HIV capsid and are unable to restrict virus replication unless encapsidated into budding virions. **(B)** Detailed sketch of Vif-mediated polyubiquitination of A3G. Vif interacts with Elongin C (EloC), which forms an obligate heterodimer with Elongin B (EloB), and Cul5. The transcription cofactor CBFβ stabilizes Vif. Cul5 binds to Rbx2and subsequently recruits an E2 ubiquitin conjugating enzyme. Vif is the substrate receptor that recruits A3 enzymes. The ^48^K-linked ubiquitin chains result in proteasomal degradation of the A3. **(C)** Sketch demonstrating the limited vulnerability of single-stranded (-)DNA to A3-mediated deamination that is imposed from the dynamics of reverse transcription. Reverse transcriptase is abbreviated as RT. HIV contains two polypurine tracts (PPT) that are used as primers for (+)DNA synthesis. In the figure, only one PPT is depicted. **(D,E)** Sketches depicting the stoichiometry of major virion components for a **(D)** WT and **(E)** ΔVif HIV virion. Figures correspond to **(D)** and **(E)** in **(A)**. **(D)** Low amounts of A3 may escape Vif-mediated degradation and become virion encapsidated (approximately one to two molecules of A3G/virion). **(E)** A ΔVif HIV cannot induce degradation of A3 enzymes and that results in the encapsidation of A3 enzymes through an interaction with RNA and Gag. Approximately 3–11 molecules of A3G can become virion encapsidated. **(D,E)** Stoichiometry values for virions were obtained from Camaur and Trono (1996), Fouchier et al. (1996), Coffin et al. (1997), Xu et al. (2007), Nowarski et al. (2008).

For HIV-1 (referred to as HIV) to successfully infect humans, it must overcome numerous physical and immunological barriers (Harris et al., 2012; Shaw and Hunter, 2012; Xu et al., 2013). Within cells, HIV must overcome a network of restriction factors that are able to block specific replication steps of the virus, including A3 enzymes (Harris et al., 2012; Rahm and Telenti, 2012). HIV can overcome these restriction factors through mutations or encoding accessory proteins that specifically block the restriction factor function (Harris et al., 2012; Rahm and Telenti, 2012). HIV uses the viral infectivity factor (Vif) to overcome A3 enzymes (Sheehy et al., 2002; Conticello et al., 2003; Harris et al., 2003; Mangeat et al., 2003; Mariani et al., 2003). The Vif protein of simian immunodeficiency virus (SIV), the non-human primate form of the virus, has co-evolved with species-specific A3s for millions of years (Compton and Emerman, 2013). The HIV-1 predecessor, SIV_cpz_ from chimpanzees underwent a key evolutionary event that altered the 3′ region of the *vif* gene that was essential for antagonism of human A3 function, along with further evolutionary changes in chimpanzees that adapted SIV_cpz_ for improved infection of humans (Etienne et al., 2013). To antagonize A3 enzymes, HIV Vif must maintain the ability to physically interact with relevant A3s, host protein CBFβ for stability (Jager et al., 2012; Zhang et al., 2012), and components of the host ubiquitin ligase assembly (Yu et al., 2003, 2004; Kobayashi et al., 2005; Xiao et al., 2006). Ultimately Vif thwarts A3s by inducing their polyubiquitination and degradation through the proteasome (**Figures 2A,B**). It is thought that by disrupting the Vif–host cell interactions through novel pharmaceuticals, A3 enzymes can be used to suppress HIV. However, the natural balance of A3 enzymes and HIV must be first understood since there is evidence that HIV can take advantage of A3 enzymes to accelerate its quasispecies evolution (Simon et al., 2005; Kim et al., 2010).

From cell culture studies, it appears that only A3A, A3D, A3F, A3G, and A3H are relevant to HIV restriction (Berger et al., 2011; Hultquist et al., 2011; Koning et al., 2011; Refsland et al., 2012; Chaipan et al., 2013). It is not surprising that not all seven A3 members restrict HIV replication since they likely evolved to restrict different retroelement pathogens (LaRue et al., 2008). There are two paradigms of how A3 enzymes can suppress HIV. A3A present in HIV target myeloid cells can restrict replication of incoming virions through low levels of deamination and possibility another mechanism that is not yet fully elucidated (Berger et al., 2011; Koning et al., 2011). In CD4+ T cells, A3D, A3F, A3G, and A3H restrict HIVΔ*vif* by becoming virion encapsidated in the HIV producer cell and traveling with the virion to the next susceptible cell where they catalyze promutagenic deaminations of cytosine to uracil in nascent single-stranded HIV (-)DNA (Hultquist et al., 2011, **Figure 2A**). Although A3B can also restrict HIV in this manner in 293T or HeLa cells, it is unable to become virion packaged and restrict HIV in T cell lines, has low expression in activated T cells, and is not antagonized by Vif, suggesting that restriction by A3B is not physiologically relevant (Doehle et al., 2005; Koning et al., 2009; Refsland et al., 2010; Hultquist et al., 2011; Pak et al., 2011). This review will focus on the restriction of HIV by virion encapsidated A3D, A3F, A3G, and A3H and how Vif antagonizes their function.

## A3-MEDIATED RESTRICTION OF HIV

The A3D, A3F, A3G, and A3H molecules that escape Vif-mediated inhibition can restrict HIV by entering the assembling virus particle by binding RNA (HIV genome or cellular RNA such as 7SL or Y) that also interacts with the nucleocapsid (NC) portion of the Gag polyprotein (Alce and Popik, 2004; Cen et al., 2004; Douaisi et al., 2004; Svarovskaia et al., 2004; Burnett and Spearman, 2007; Khan et al., 2007; Bach et al., 2008; Bogerd and Cullen, 2008; Strebel and Khan, 2008; Wang et al., 2008; Ooms et al., 2010; Zhen et al., 2012, **Figure 2A**). After the virus enters the next target cell A3 enzymes exert their anti-viral function during the reverse transcription process (Suspene et al., 2004; Yu et al., 2004; Zheng et al., 2004; Dang et al., 2006; OhAinle et al., 2008, **Figures 2A,C**). Although A3D, A3F, A3G, and A3H are localized to the cytoplasm they require encapsidation to restrict HIV and are not able to access the (-)DNA of an incoming virus (Hultquist et al., 2011, **Figure 2A**). This may be due to the HIV capsid structure or that A3 enzymes can reside in regions of RNA processing, e.g., stress granules or P-bodies, where they may have a role in sequestering human retrotransposon RNA to prevent transposition (Chiu et al., 2006; Kozak et al., 2006; Stopak et al., 2007; Gallois-Montbrun et al., 2008). Since A3 enzymes are ssDNA deaminases, deamination activity is restricted to the (-)DNA strand (Yu et al., 2004, **Figure 2C**). The cytosine (C)→uracil (U) deaminations catalyzed on the (-)DNA strand become guanine (G)→adenine (A) mutations when reverse transcriptase (RT) uses U as a template during (+)DNA strand synthesis (Yu et al., 2004, **Figure 2C**). The resulting “hypermutation” of the provirus leads to inactivation of HIV (Harris et al., 2003; Mangeat et al., 2003; Zhang et al., 2003, **Figure 2A**). Although it is known that many proviral genomes undergo successful integration with these hypermutations (Russell et al., 2009a), some preintegration complexes containing U may be degraded by host DNA repair mechanisms, although there is no consensus regarding the extent to which this occurs in cells (Kaiser and Emerman, 2006; Yang et al., 2007). In cell culture, it has been found that the mutated HIV proteins that may be produced from these proviral genomes can act as a source of HIV antigens due to their misfolding and processing through the proteasome, which can facilitate immune recognition of HIV (Casartelli et al., 2010).

Each ssDNA deaminase acts within a preferred di- or tri- nucleotide substrate motif. For A3G, this is 5′CCC or 5′CC (Yu et al., 2004, 3′-end C preferred for deamination). A3D, A3F, and A3H deaminate 5′TTC or 5′TC motifs and A3D can also deaminate 5′GC motifs in proviral DNA (Liddament et al., 2004; Dang et al., 2006; Ooms et al., 2013a). Although the majority of A3 actions are repressed by Vif in HIV-infected individuals (**Figures 2A,B,D**), clinical studies have found that individuals with an inherent ability to express a high level of A3G mRNA are less likely to become infected with HIV or progress from HIV to AIDS and that the presence of hypermutated proviral genomes correlates with high CD4+ T-cell counts (Jin et al., 2005; Pace et al., 2006; Biasin et al., 2007; Land et al., 2008; Vazquez-Perez et al., 2009). Other A3 enzymes have not been extensively examined in this regard (Albin and Harris, 2010; Ooms et al., 2013a). However, there is evidence of deaminations in HIV genomes recovered from infected individuals due to C/G→T/A mutations in a sequence context that indicates deaminations by A3 enzymes other than A3G do occur (Pace et al., 2006; Land et al., 2008; Vazquez-Perez et al., 2009; Ooms et al., 2013a). For example, a study found that in HIV-infected individuals there is approximately an equal split between mutations occurring in the 5′TC and 5′CC contexts on (-)DNA (Ooms et al., 2013a). However, there is difficulty parsing out the effect of A3F, A3D, and A3H based on their mutation patterns since they all recognize the minimal dinucleotide 5′TC and are more promiscuous than A3G in regard to trinucleotide target site preference (Liddament et al., 2004; Dang et al., 2006; Hultquist et al., 2011). Nonetheless, it has become clear that despite some evidence that A3G has more mutagenic potential than other A3 deaminases, it is not acting alone against HIV (Refsland et al., 2012; Chaipan et al., 2013; Ooms et al., 2013a). The HIV genomes mutated through A3 catalytic activity are also subject to the pressure of purifying selection. This selection pressure results in mutated and inactivated genomes being highest in integrated proviral DNA and lowest in circulating viral RNA (Russell et al., 2009a). Furthermore, integrated proviruses that are inactivated by stop codons in the *gag* gene may still be rescued by dual infection of a cell by HIV quasispecies and complementation of Gag function (Russell et al., 2009a). Recombination within virions by RT template switching can result in “reactivation” of inactivated viral genomes (Mulder et al., 2008; Russell et al., 2009a). As a result, A3-mediated mutagenesis is effective, but the complete inactivation of HIV in an infected individual is potentially a long-term process that is likely to require multiple rounds of exposure to viruses.

### A3G-MEDIATED RESTRICTION OF HIV

#### Deamination-dependent HIV restriction by A3G

Since A3G was the first A3 enzyme discovered A3G has been the most widely studied for how it enacts its role as a restriction factor (over 700 publications in PubMed). There are two key steps that A3G must complete to be an efficient restriction factor. First, the enzyme must be available for binding RNA that will become virion encapsidated through an interaction with the NC portion of Gag (Alce and Popik, 2004; Cen et al., 2004; Douaisi et al., 2004; Svarovskaia et al., 2004; Burnett and Spearman, 2007; Khan et al., 2007; Bach et al., 2008; Bogerd and Cullen, 2008; Strebel and Khan, 2008; Wang et al., 2008). Second, it must have a mechanism to search the nascent HIV (-)DNA, that is available for a finite period of time, for potential cytosines that it can deaminate (Chelico et al., 2006; Nowarski et al., 2008; Ara et al., 2014, **Figure 2C**).

**FIGURE 3:**
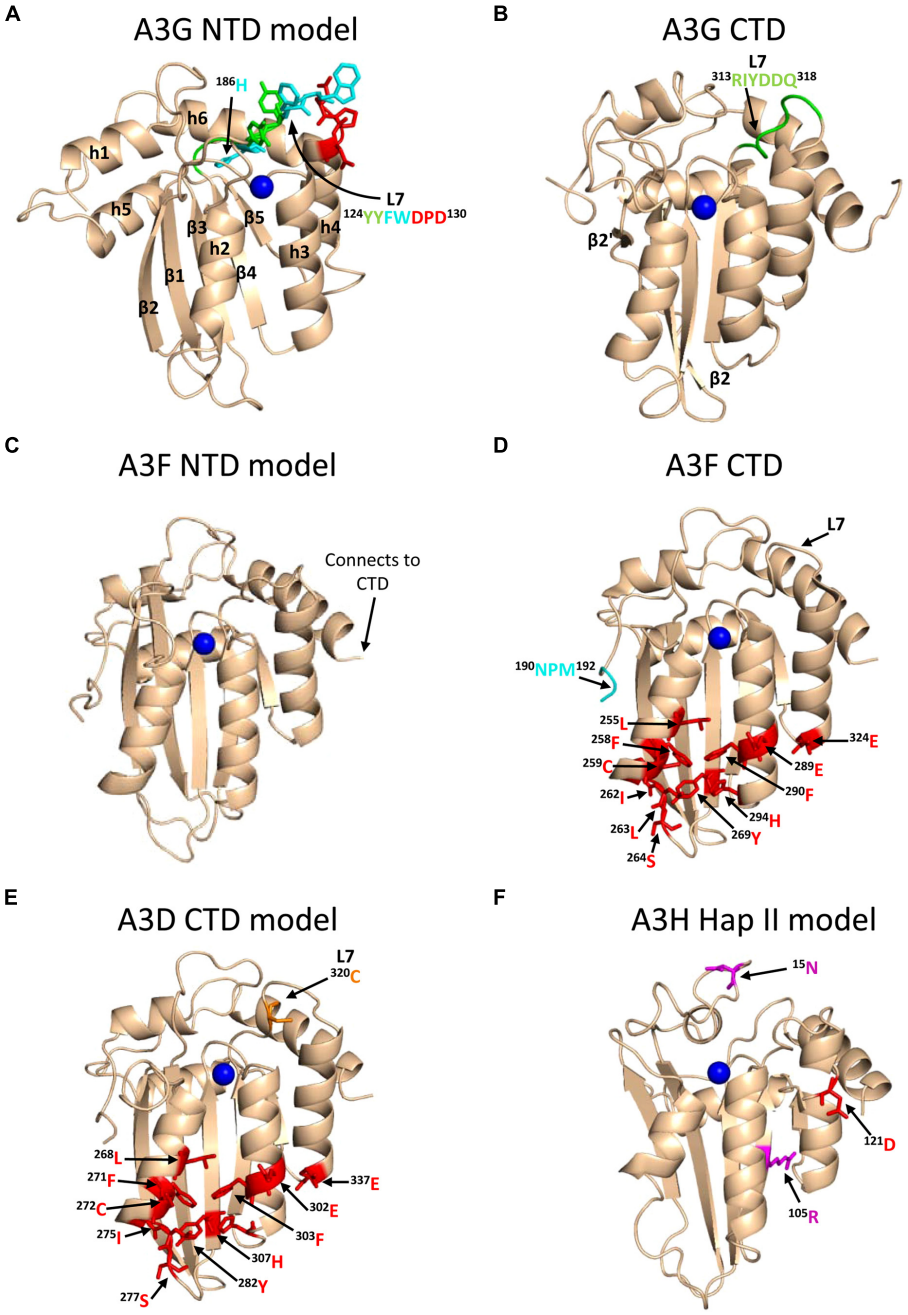
**Structures of A3 enzymes.** A3 enzymes have a basic structure in each Z-type domain that is composed of a five-stranded β-sheet core surrounded by six α-helices. Numerical assignments to β-strands and α-helices are superimposed in **(A)**. Zinc atoms are shown as blue spheres. **(A)** Model of the N-terminal domain (NTD) of A3G. Loop 7 (L7) of the A3G NTD is a central structure in its anti-HIV function. Highlighted on L7 are the residues important for interaction with Vif (red, ^128^DPD^130^), oligomerization/virion encapsidation (green and cyan, ^124^YYFW^127^), and jumping component of A3G processivity (cyan, ^126^FW^127^). Helix 6 (h6) is adjacent to L7 and contributes to the sliding component of A3G processivity, particularly ^186^H (cyan). The model of the A3G NTD was obtained by using the automated SWISS-MODEL program using the homologous A3G C-terminal domain structure (CTD, PDB: 3IQS). **(B)** The A3G CTD (PDB: 2KEM) is the catalytic domain of A3G. The A3G CTD has a discontinuous β2 strand forming a loop-like bulge between the β2 and β2′ strands. A3G L7 residues ^313^RIYDDQ^318^ (green) mediate tetramerization and determine the preferred deamination motif. **(C)** The model of the A3F NTD was obtained by using the automated SWISS-MODEL program using the homologous A3C structure (PDB: 3VM8). The end of h6 connects the NTD to the CTD and contains an ^190^NPM^192^ motif. This NPM motif is found only in A3D and A3F. **(D)** The A3F CTD (PDB: 4IOU) is the catalytic domain of A3F and interacts with Vif. Residues that interact with Vif across Helix 2, 3, 4, and β-strand 4 are shown in red. Also shown on this structure is the deamination motif specificity loop (L7) and the ^190^NPM^192^ motif. The structure illustrates the kinked orientation introduced by the Pro in the ^190^NPM^192^ motif, which blocks the sliding function of A3F. **(E)** The model of the CTD of A3D was obtained by using the SWISS-MODEL program using the homologous A3F structure (PDB: 4IOU). Residues that interact with Vif across Helix 2, 3, 4, and β-strand 4 are shown in red. The ^320^C residue on L7 that influences A3D activity is shown in orange. **(F)** Model of A3H Hap II showing residues that interact with Vif and cause haplotype instability. In A3H Hap II, ^121^D (red) on predicted h4 mediates an interaction with Vif. In A3H Hap I the R105G mutation induces protein instability (magenta). In A3H Hap III and IV, the deletion of ^15^N induces protein instability (magenta). The model of the A3H Hap II was obtained by using the automated SWISS-MODEL program using the homologous APOBEC2 structure (PDB: 2NYT). Figures were made using PyMOL (The PyMOL Molecular Graphics System, Version 1.5.05, Shrödinger, LLC.).

A3G exists in cells as a high molecular mass that is bound to RNA and other proteins in stress granules and P-bodies (Chiu et al., 2006; Kozak et al., 2006; Wichroski et al., 2006; Gallois-Montbrun et al., 2008). However, only newly synthesized A3G that has not associated with host RNAs in these cytoplasmic structures appears to bind the RNA that is also bound by HIV Gag and therefore encapsidated into virions (Soros et al., 2007). A3G requires oligomerization to bind these RNAs effectively in cells and become virion encapsidated (Wang et al., 2007; Bulliard et al., 2009; Huthoff et al., 2009), but *in vitro* oligomerization mutants of A3G can bind many RNAs with less than a threefold difference from wild-type (WT) A3G (Chelico et al., 2010; Feng and Chelico, 2011). The RNA binding and oligomerization of A3G is primarily mediated by the N-terminal domain (NTD) and the NTD is solely responsible for virion encapsidation of A3G (Hache et al., 2005; Navarro et al., 2005; Huthoff et al., 2009; Chelico et al., 2010, **Figure 1**). The NTD residues ^124^YYFW^127^ on predicted loop 7 mediate the dimerization of A3G (Huthoff et al., 2009; Chelico et al., 2010, **Figure 3A**). A3G is primarily a dimer in solution and when A3G binds RNA or DNA it oligomerizes into tetramers and higher order structures through C-terminal domain (CTD) residues ^313^RIYDDQ^318^ on loop 7 (Chelico et al., 2008, 2010; Shlyakhtenko et al., 2011, **Figure 3B**). It is essential for A3G to enter the inner capsid of the virion to restrict HIV. Within the capsid A3G can associate with the ribonucleoprotein complex and access the (-)DNA as it is synthesized. A3 enzymes that cannot encapsidate within the HIV capsid, e.g., A3A and A3C, are unable to restrict HIV replication in CD4+ T cells (Goila-Gaur et al., 2007; Hultquist et al., 2011). However, accessing the ribonucleoprotein complex does not guarantee the ability to restrict HIV. Since the (-)DNA is only available for a finite period of time due to HIV containing two polypurine tracts (PPT) that are used to prime (+)DNA synthesis (Suspene et al., 2006; Hu et al., 2010), A3 enzymes require an efficient mechanism to search for cytosines (**Figure 2C**). Complicating the search is that the (-)DNA contains pieces of annealed RNA due to the endonuclease activity of the RT-associated RNaseH (**Figure 2C**). A3G binds RNA/DNA hybrids less well than ssDNA and encountering these obstacles on the substrate can induce A3G to dissociate from DNA (Iwatani et al., 2006; Chelico et al., 2008).

**FIGURE 4:**
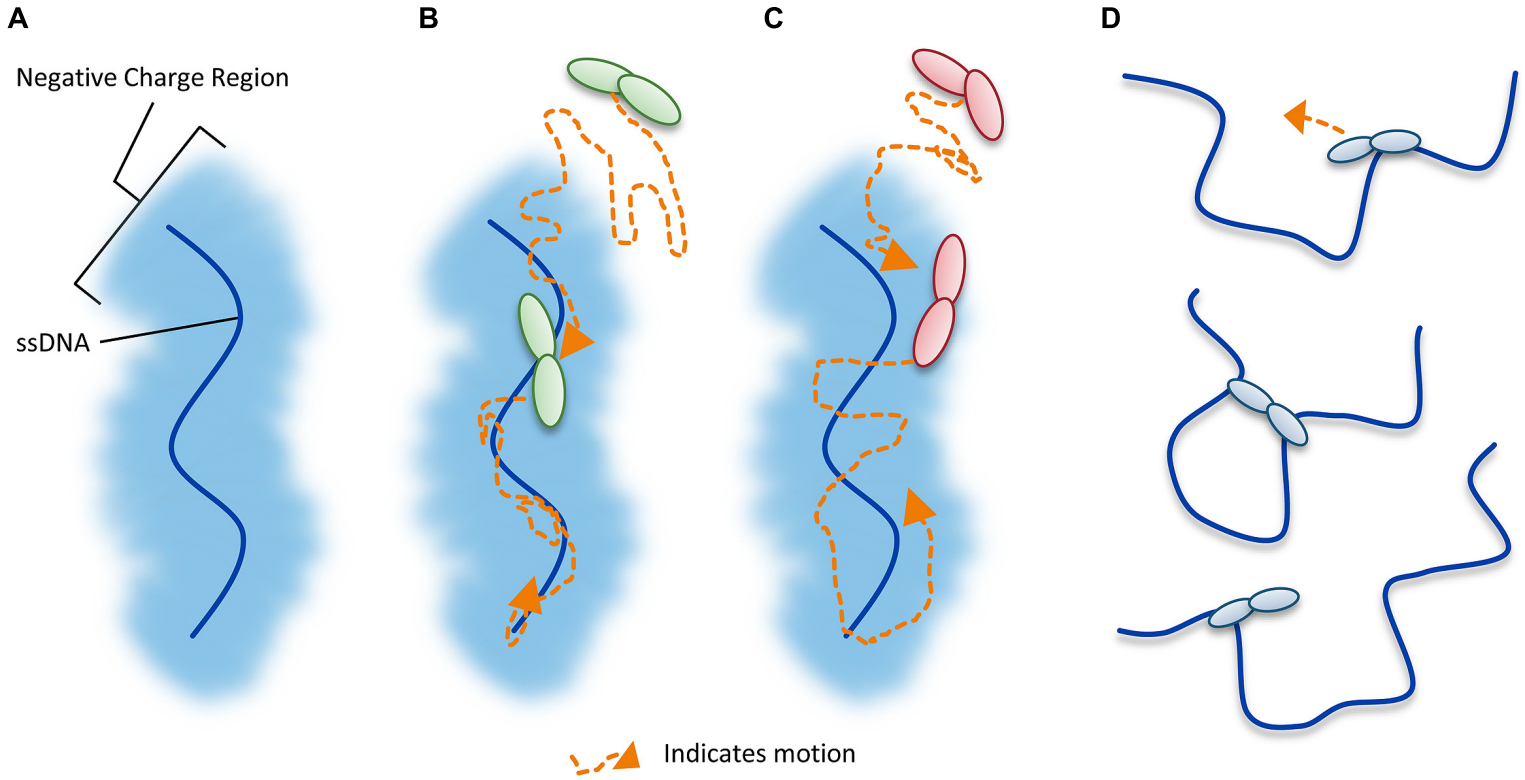
**Illustration of DNA scanning by facilitated diffusion. (A)** Sketch of DNA showing the negatively charged region of DNA important for facilitated diffusion of A3 enzymes. **(B–D)** Enzyme in sketches is shown as a dimer, although the oligomerization state may vary with different A3 enzymes. **(B)** Sketch depicting a 1-dimensional DNA scanning path by sliding. Dotted line indicates path of enzyme (orange). Sliding enables an in depth search of local areas of a substrate. **(C)** Sketch depicting a 3-dimensional DNA scanning path by jumping. Jumping enables larger translocations on DNA substrates, but lacks a local search process. The microdissociations of the enzyme from the DNA that occur when the enzyme jumps does not leave the negatively charged domain of the DNA so the enzyme has a higher likelihood of reassociating with the same DNA substrate than diffusion into the bulk solution. **(D)** Sketch depicting a 3-dimensional DNA scanning path by intersegmental transfer. Intersegmental transfer enables larger translocations on DNA substrates, but lacks a local search process. An enzyme with two DNA-binding domains binds two regions of DNA simultaneously before dissociating from one region to move to another.

Unraveling the mechanism by which A3G locates and catalyzes its deamination motif is of pivotal importance for understanding the mechanistic basis of proviral hypermutation. The mechanism with which an A3 enzyme scans non-target DNA in search of its deamination motif is a determinant in its catalytic efficiency (Feng and Chelico, 2011; Ara et al., 2014). DNA scanning is described by the term processivity and is defined as the ability of an enzyme to catalyze multiple events in a single enzyme–DNA substrate encounter. Enzymes that do not use an energy source for movement on DNA use a mechanism termed facilitated diffusion to efficiently search DNA (Berg et al., 1981; von Hippel and Berg, 1989). This is a mechanism where the enzymes, subject to Brownian motion, move randomly on DNA. Since DNA-binding proteins are usually positively charged, the negative charge of the DNA facilitates the enzyme movement through electrostatic interactions (Berg et al., 1981; von Hippel and Berg, 1989). A3G is a positively charged enzyme (charge of +6.5 at pH 7) and processively scans ssDNA by facilitated diffusion (Chelico et al., 2006; Nowarski et al., 2008, **Figure 4**). This mode is distinct from an enzyme that acts on DNA distributively, where only one catalytic event occurs before the enzyme disengages from the substrate (Chelico et al., 2009). Facilitated diffusion can involve a variety of movements such as 1-dimensional (D) sliding (**Figure 4B**) or 3-D movements such as hopping/jumping (**Figure 4C**) or intersegmental transfer (**Figure 4D**, Halford and Marko, 2004). Hopping and jumping describe small microdissociations and reassociations with the same DNA strand without diffusion into the bulk solution (von Hippel and Berg, 1989, **Figure 4C**). Intersegemental transfer involves a two-step mechanism where an enzyme with two DNA-binding sites binds a second site before releasing the first site (von Hippel and Berg, 1989, **Figure 4D**). Facilitated diffusion works best when both 1- and 3-D movements are used to enable local scanning of a small segment of DNA by sliding (<20 nt) and movement to distal regions to restart the local scanning process (Halford and Marko, 2004; Feng and Chelico, 2011). These distal movements do not cause the enzyme to leave the DNA and enter into the bulk solution because the charged surface of the DNA keeps the enzyme within the domain of the DNA (von Hippel and Berg, 1989, **Figure 4C**). Using different methods A3G has been found to scan ssDNA by 1-D sliding motions and 3-D jumping motions (Chelico et al., 2006; Senavirathne et al., 2012; Shlyakhtenko et al., 2012). However, one study has found that A3G moves by 3-D intersegmental transfers (Nowarski et al., 2008). The efficiency imparted by a 3-D movement in the specific case of A3G during reverse transcription is that it provides a means of overcoming the DNA/RNA hybrid barrier (Nowarski et al., 2008; Feng and Chelico, 2011). Clusters of A3G-induced deaminations indicative of processive sliding movements have been found in integrated proviral genomes (Browne et al., 2009) and A3G mutants unable to undergo a local searching process by 1-D sliding, such as H186R and A3G with an ^195^NPM^197^ insertion have decreased mutagenesis during *in vitro* reverse transcription or in HIV proviral genomes (Feng and Chelico, 2011; Ara et al., 2014). Furthermore, an A3G F126A/W127A mutant that cannot jump has decreased mutagenesis during *in vitro* reverse transcription (Feng and Chelico, 2011). These data demonstrate that neither movement alone can enable high levels of A3G-induced mutagenesis. Interestingly, the F126A/W127A mutant is monomeric, suggesting that the oligomeric state of A3G plays a role in efficient restriction of HIV not only by facilitating virion encapsidation but also by facilitating the DNA scanning process (Huthoff et al., 2009; Chelico et al., 2010; Feng and Chelico, 2011). The processivity determinants of A3G reside on predicted loop 7 and helix 6 of the non-catalytic NTD (Feng and Chelico, 2011; Ara et al., 2014, **Figure 3A**). Thus, despite a lack of catalytic activity, the NTD contributes to A3G deamination activity by mediating the processive scanning mechanism (Feng and Chelico, 2011).

#### Deamination-independent HIV restriction by A3G

A3G primarily restricts HIV replication through its deamination activity. However, there have been numerous reports of an ability of A3G to physically inhibit other processes of HIV such as RT polymerization (Iwatani et al., 2007; Bishop et al., 2008; Wang et al., 2012; Adolph et al., 2013; Belanger et al., 2013; Gillick et al., 2013), NC strand annealing (Guo et al., 2006, 2007), and proviral DNA integration (Luo et al., 2007; Belanger et al., 2013). These processes do not occur in isolation from deamination, nor do they restrict HIV better than deamination alone (Belanger et al., 2013; Gillick et al., 2013). We will focus on inhibition of RT polymerization since this is the most prevalent mechanism studied.

Early studies of A3G-mediated restriction of HIV proposed that transiently overexpressed WT A3G and deamination null mutants of A3G could inhibit HIV proviral DNA formation (Mangeat et al., 2003; Newman et al., 2005). The initial mechanism proposed was that A3G binds the HIV genomic RNA which impedes RT (Iwatani et al., 2007). This has been confirmed in multiple reports using cellular and biochemical experiments (Adolph et al., 2013; Belanger et al., 2013; Chaurasiya et al., 2014). However, the physiological significance of these processes is difficult to reconcile since results from cell-based experiments using transiently expressed A3G cannot be replicated when A3G is stably expressed, suggesting that overexpression of A3G induced experimental artifacts due to excessive packaging of A3G in virions (Mbisa et al., 2007; Miyagi et al., 2007; Schumacher et al., 2008; Browne et al., 2009). Importantly, studies that used deamination null mutants of A3G to show that deamination ability is required for restriction of HIV (Mbisa et al., 2007; Miyagi et al., 2007; Schumacher et al., 2008) should be considered in conjunction with data showing that the A3G E259Q catalytically inactive mutant is not a true proxy for A3G (Bishop et al., 2008; Adolph et al., 2013). A3G E259Q binds RNA less well than A3G and this results in less inhibition of RT *in vitro* and in cells (Bishop et al., 2008; Adolph et al., 2013).

Nonetheless, it is clear that the ability of A3G to inhibit RT is highly dependent on A3G concentration and the primer/template (Mbisa et al., 2007; Miyagi et al., 2007; Schumacher et al., 2008; Browne et al., 2009; Adolph et al., 2013). The initiation of DNA synthesis from an RNA primer on an RNA template is the least efficient type of polymerization activity of RT (Liu et al., 2010). Accordingly, *in vitro*, low levels of A3G can best inhibit RT-mediated primer initiation at this step by competing for substrate (Iwatani et al., 2007; Adolph et al., 2013). In contrast, on a DNA primer and DNA template, A3G could at most inhibit *in vitro* RT-mediated initiation of synthesis by twofold under single turnover conditions and could not block RT from binding the primer/template, but merely lengthened the time RT required to find the free 3′OH (Adolph et al., 2013). These data are in agreement with a computational study that suggests an A3G-mediated deamination-independent mode of HIV restriction contributes <1% of the restriction capability of A3G (Kobayashi et al., 2014). Although studies have shown that a peptide of A3G can interact with RT and inhibit RT-mediated DNA synthesis, it is unlikely that this mechanism is a physiological way to inhibit reverse transcription since in ΔVif virions, only 3–11 A3G molecules are encapsidated whereas there are approximately 100 RT molecules (Coffin et al., 1997; Xu et al., 2007; Wang et al., 2012, **Figure 2E**). In the presence of Vif, there is only an estimated one to two molecules of A3G per virion (Nowarski et al., 2008, **Figure 2D**), emphasizing the importance of a deamination-dependent mechanism over a deamination-independent mechanism. A single molecule of A3G could inactivate an HIV provirus through cytosine deamination whereas the deamination-independent mechanism is much more concentration dependent (Browne et al., 2009; Adolph et al., 2013). Single-molecule studies have brought forth the model that A3G oligomers can act as a road-block for HIV (Chaurasiya et al., 2014). Notably, existence of a deamination-independent mode of HIV inhibition has been observed *ex vivo* in primary cells (Gillick et al., 2013), but it requires further research as to the significance of this mode of inhibition during an HIV infection.

### A3F-MEDIATED RESTRICTION OF HIV

#### Deamination-dependent HIV restriction by A3F

Approximately 2 years after the discovery of A3G, A3F was discovered to also exhibit restriction activity against HIV (Liddament et al., 2004; Wiegand et al., 2004; Zheng et al., 2004). Sequenced HIV proviral genomes were known to contain G/C→A/T transition mutations in 5′CC and 5′TC contexts in the (-)DNA (Pathak and Temin, 1990; Li et al., 1991; Vartanian et al., 1991, 2002; Fitzgibbon et al., 1993) and A3F was found to contribute to transition mutations in the 5′TC context. Of these initial studies demonstrating A3F activity active against HIV (Bishop et al., 2004; Liddament et al., 2004; Wiegand et al., 2004; Zheng et al., 2004), all except one (Zennou and Bieniasz, 2006) found that A3F restriction activity was equivalent to A3G restriction activity. It has since been shown that overexpression of A3 enzymes can result in excessive packaging into HIV virions and result in artifacts of HIV restriction (Miyagi et al., 2007; Xu et al., 2007; Schumacher et al., 2008). Yet even after 10 years of studying A3F, multiple groups still find different activities of A3F against HIV that cannot be attributable to overexpression, but perhaps different experimental systems and techniques (Miyagi et al., 2010; Mulder et al., 2010; Hultquist et al., 2011; Chaipan et al., 2013; Ara et al., 2014). However, A3F must exert a restriction pressure on HIV since Vif maintains an interaction interface with A3F that is distinct from A3G in order to induce A3F degradation (Russell et al., 2009b). As with A3G, for A3F to effectively restrict HIV, it must be encapsidated with the ribonucleoprotein complex within the capsid (Wang et al., 2008) and effectively search for cytosines on the heterogeneous (-)DNA substrate (Ara et al., 2014).

A3F encapsidates into HIV virions through an interaction with RNA, but packages more efficiently than A3G into the core of HIV particles (Zennou and Bieniasz, 2006; Wang et al., 2008; Song et al., 2012). By resolving HIV capsids on a sucrose gradient to observe whether A3F and A3G partition with the RNA and enzymes or the p24 capsid protein, Song et al. (2012) found that more A3F specifically associated with the ribonucleoprotein complex, in comparison to A3G. A3F binds nucleic acids with sevenfold higher affinity than A3G (Ara et al., 2014), which may enable it to package more specifically within the capsid (Song et al., 2012). Furthermore, A3F has been shown to bind double-stranded DNA with a higher affinity than A3G and maintain an association with the pre-integration complex of HIV as it enters the nucleus through its high-affinity nucleic acid binding (Mbisa et al., 2010; Burdick et al., 2013). Despite the quantity of A3F being at an equal or greater amount to A3G, A3F restricted HIV approximately fourfold less than A3G in a single round of infection (Song et al., 2012). Although some reports show A3F can be less effective than A3G in restricting HIV (Miyagi et al., 2010; Mulder et al., 2010; Song et al., 2012; Chaipan et al., 2013; Ara et al., 2014), it cannot be concluded that it does not suppress HIV or impose selective pressure on HIV. Not only because of data showing A3F can effectively restrict HIV in spreading infections (Hultquist et al., 2011; Refsland et al., 2012), but also because an HIV lab strain with tandem stop codons in Vif (from HIV NL4-3) will revert back to expressing a functional Vif in the presence of A3F (Albin et al., 2010a). This does not occur when A3G is used in the same type of forced evolution experiments (Hache et al., 2008, 2009). The HIV evolves to overcome A3G restriction, but does so by acquiring a 5′UTR mutation to make HIV RNA transcription more efficient and altering the cell cycle through a Vpr mutation (Hache et al., 2008, 2009). Together these mutations result in more virus particles being produced. Presumably since A3G has less specific packaging in the capsid than A3F, this strategy titrates out the ribonucleoprotein-packaged A3G enabling the HIV to escape high levels of mutagenesis. These data illustrate that A3G and A3F exert a distinct selective pressure on HIV due to distinct biochemical properties and that A3 packaging into virions is a necessary but insufficient step to ensure efficient HIV restriction (Ara et al., 2014).

To further understand why A3F-mediated restriction of HIV may be different than for A3G, Ara et al. (2014) undertook a biochemical study of A3F in comparison to A3G to identify biochemical differences between these enzymes that could account for differences in restriction efficiency. They found that in contrast to A3G, A3F used only 3-D jumping motions to scan ssDNA. This made the DNA scanning mechanism inefficient since A3F could translocate between many ssDNA regions and overcome intervening RNA/DNA hybrid regions, but lacked a local search mechanism to examine ssDNA regions for its 5′TC motif (**Figure 4C**). The A3F sliding movement is blocked by a ^190^NPM^192^ motif in the connection domain between the NTD and CTD (**Figures 3C,D**) since mutagenesis of this motif to ^190^NGM^192^enabled A3F to slide (Ara et al., 2014). The Bohn et al.’s (2013) A3F CTD structure includes the ^190^NPM^192^ sequence and shows that it is a kinked region of the loop structure (**Figure 3D**). Since an A3F ^190^NGM^192^ mutant was able to slide, the data suggest that the rigid ^191^P residue primarily blocks sliding. However, imparting sliding movement to A3F through the ^190^NGM^192^ mutant did not increase A3F HIV restriction efficiency because the jumping movements of A3F differed from A3G and were dominant over sliding which maintained an inefficient search of ssDNA (Ara et al., 2014). The differences in DNA scanning between A3F and A3G were shown to be relevant to HIV restriction since A3F was fourfold less effective in restriction of HIV than A3G in a single-cycle infectivity assay (Ara et al., 2014). Of note, A3F was also shown to have a 100-fold lower specific activity than A3G (Ara et al., 2014), but this was not thought to contribute to differences in restriction efficiency since studies with different A3G and A3F mutants showed that mutagenesis efficiency correlated with the efficiency of the ssDNA scanning mechanism, not the specific activity. This is likely because RT polymerization and RNaseH activity limit the (-)DNA substrate available (Feng et al., 2013). The study by Ara et al. (2014) is in agreement with studies where A3F has not been as effective as A3G in restriction of HIV (Miyagi et al., 2010; Mulder et al., 2010; Chaipan et al., 2013), although A3F was found to be as restrictive to HIV replication as A3G in other reports (Hultquist et al., 2011; Refsland et al., 2012). Despite A3F being considered in some reports to be less efficient than A3G as an HIV restriction factor when considered side by side, this is far from the natural mechanism of these enzymes in which they act in concert (Refsland et al., 2012; Ooms et al., 2013a) and further studies examining how these enzymes work together are needed.

It is of note that Zennou and Bieniasz (2006) noticed that per mutation, A3F was less likely to inactivate HIV than A3G. This was later found to be because the 5′CC motif of A3G overlaps with the only Trp codon (5′TGG/ACC) and results in a stop codon upon deamination of either cytosine in the motif (Yu et al., 2004). In contrast, A3F-induced mutations largely result in missense mutations which may or may not inactivate the encoded protein (Ara et al., 2014). The A3G 5′CC motif also overlaps with Gly codons and in the HIV *prot* mutations at these Gly results in more non-conservative mutations and gene inactivation than A3F-induced missense mutations that primarily cause the conservative mutation of Glu to Gln (Ara et al., 2014). The determinant for motif specificity is loop 7 in the CTD (Langlois et al., 2005; Kohli et al., 2009; Carpenter et al., 2010; Rathore et al., 2013, **Figures 3B,D**). This loop can be grafted into different A3 enzymes to change site specificity (Kohli et al., 2009). However, the consequences of deamination mediated restriction can be independent from inducing amino acid changes. A3F and A3G may be able to block proviral integration through deoxycytidine deaminations that result in aberrant processing of the proviral DNA ends by HIV integrase and inhibition of plus-strand DNA transfer by reducing the efficiency of primer tRNA removal (Mbisa et al., 2007, 2010).

#### Deamination-independent HIV restriction by A3F

For many years, A3F was thought to have a stronger deamination-independent mode of inhibiting HIV than A3G (Bishop et al., 2006; Holmes et al., 2007). Unlike A3G, the mechanism of deamination-independent “activity” was not extensively studied, but was presumed to be due to inhibition of RT polymerization. A computational study has found that A3G and A3F rely differentially on their deamination-independent modes of HIV restriction with A3G only having the deamination-independent mode contributing to <1% of its restriction activity whereas for A3F this value was approximately 30% (Kobayashi et al., 2014). However, two studies using stably expressed A3F and A3F catalytic mutants C280S/C283A and E251Q demonstrated no inhibition of RT, suggesting that previous results were influenced by A3F overexpression artifacts (Miyagi et al., 2010; Albin et al., 2014). Another study showed that A3F can inhibit HIV integration by reducing 3′ processing of viral DNA at the U5 and U3 ends by integrase (Mbisa et al., 2010). Using a catalytic mutant of A3F (E251Q), the study found that inhibition of integration was decreased approximately twofold from that of WT A3F suggesting that catalytic activity is in part required to produce the aberrant U5 and U3 ends (Mbisa et al., 2010). Thus there appears to be consensus that despite the potentially inefficient mutagenic activity of A3F in some studies, the deamination activity of A3F is still dominant over the deamination-independent activity. Furthermore, if a deamination-independent mode of HIV inhibition functions in cells, it may be the inhibition of integration rather than reverse transcription (Mbisa et al., 2010).

### A3D-MEDIATED RESTRICTION OF HIV

A3D was first characterized in 2006 to restrict HIV replication in single-cycle infectivity assays and to be suppressed by Vif, suggesting that it posed a restriction pressure on HIV (Dang et al., 2006). Further evidence of this was that HIV proviral genomes showed evidence of deaminations in the contexts of 5′CC, 5′TC, and 5′GC (Dang et al., 2006). A3D was found to deaminate in the 5′TC and 5′GC contexts which were unique from A3G and A3F that maintain less promiscuous deamination motif preferences (Dang et al., 2006). A3D also forms multimers through an RNA intermediate in cells with a similar profile as A3G (Li et al., 2014). In a clinical study of HIV-infected individuals, A3D was found to be upregulated in both Elite Controllers and in Non-Controllers but was down-regulated in response to successful anti-retroviral treatment, indicating that A3D is part of a virological immune response to HIV (Abdel-Mohsen et al., 2013). However, the restrictive activity of A3D appears less than A3G and A3F in single-cycle infectivity assays in cell lines (Dang et al., 2006, 2011; Hultquist et al., 2011) and spreading infections in primary human cells (Chaipan et al., 2013). Furthermore, A3D represents the most divergent A3 enzyme in the lineage of chimpanzee to humans and the activity of A3D has decreased from chimpanzees to humans (Duggal et al., 2011). Other chimpanzee and human A3 enzymes are more commonly found to have similar restriction potentials (Duggal et al., 2011). Chimpanzee A3D induces more hypermutation of HIV than human A3D, despite equivalent packaging into virions (Duggal et al., 2011). This was attributed to differences in loop 7 of the CTD (Dang et al., 2011; Duggal et al., 2011). One report found a single amino acid in the CTD loop 7, C320, that suppressed A3D antiviral activity (Dang et al., 2011, **Figure 3E**). If the C320 was replaced with a Tyr, as in A3F, the activity of A3D could be increased by more than 20-fold (Dang et al., 2011). In contrast, endogenous A3D from the T cell line CEM2n appears to have activity against HIV-1 that is similar to A3F (Refsland et al., 2012). Using a series of *A3 null* backgrounds or A3 knockdowns, Refsland et al. (2012) found that the HIV-1 proviral hypermutation pattern at 5′CC and 5′TC sites was induced at comparable levels by the combined action of A3G and A3F or A3G and A3D, suggesting a redundancy in the HIV-1 restriction mechanism.

### A3H-MEDIATED RESTRICTION OF HIV

A3H was originally identified as not being able to restrict HIV replication due to low steady-state protein levels in mammalian cells, despite normal mRNA expression (Dang et al., 2006; OhAinle et al., 2006). However, when A3H was recombinantly expressed in *Escherichia coli* it could mutate the *E. coli* genomic DNA (OhAinle et al., 2006). In later studies, it was realized that A3H exists as multiple haplotypes in the human population (Hap I-VII) with different stabilities in cells and HIV restriction capabilities (**Table 1**) and the original A3H tested was an unstable form (Hap I, OhAinle et al., 2008; Harari et al., 2009). The unstable Hap I is the most prevalent form of A3H in the population (**Table 1**), but is able to restrict HIV infection by approximately twofold when transiently overexpressed in cell lines (OhAinle et al., 2008; Harari et al., 2009; Li and Emerman, 2011; Wang et al., 2011a). Two amino acid polymorphisms, ^105^G and Δ^15^N, can independently contribute to the instability of A3H (**Table 1**). A3H Hap I is unstable due to a Gly at position 105 (OhAinle et al., 2008). An A3H Hap I G105R mutant (later identified as Haplotype VII, **Table 1**) renders the A3H stable in cells and imparts strong anti-HIV activity (OhAinle et al., 2008; Harari et al., 2009). Other unstable A3H haplotypes (III and IV) have the Δ^15^N in combination with another polymorphism (OhAinle et al., 2008; Harari et al., 2009, **Table 1**). It is not known biochemically why these A3H haplotypes are unstable, but comparative modeling of A3H with the structure of a related family member APOBEC2 shows that amino acid 105 is in a β-strand within the central five-stranded β-sheet, suggesting that an R105G mutation could destabilize the core structure (**Figure 3F**). The Δ ^15^N is predicted to be within a loop structure (**Figure 3F**) so it is difficult to predict the reason for the instability in this undefined region, but it is known from studies with A3F that deletions to a loop that connect the NTD and CTD cause protein instability (Ara et al., 2014), suggesting that the A3H loop may need to be of a specific length for proper protein folding. Although different haplotypes (II, V, and VII) have been reported to exist in the population as stable forms that are able to restrict HIV (**Table 1**), in this review we focus only on A3H Hap II (A3H Hap II), which has been the most highly studied. Notably, A3H Hap II has some variability in its restriction ability which is dependent on alternative mRNA spliced forms (Harari et al., 2009). A3H Hap II is primarily found in Africans/African Americans (∼50%) and to a much lesser extent within other cultural populations (prevalence of approximately 0–8%, OhAinle et al., 2008; Wang et al., 2011b). It has been proposed that A3H evolved to become unstable due to a combination of the loss of an ancient pathogen and the ability of an ancestral A3H to induce mutagenesis of genomic DNA (Jern and Coffin, 2008; OhAinle et al., 2008).

**Table 1:** Summary of A3H haplotype features.

A3H haplotypes	Polymorphic amino acid residues					Antiviral activity in cell culture	Protein stability	Haplotype frequency	Reference
	Δ15	18	105	121	178				
Hap I	N	R	G	K	E	Partial	No	0.526^a^	OhAinle et al. (2006), OhAinle et al. (2008), Dang et al. (2008), OhAinle et al. (2008), Harari et al. (2009), Ooms et al. (2010, 2013a), Li and Emerman (2011), Wang et al. (2011b), Zhen et al. (2012)
								0.308^b^	
Hap II	N	R	R	D	D	Yes	Yes	0.061^a^	OhAinle et al. (2008), Ooms et al. (2010), Hultquist et al. (2011), Li and Emerman (2011), Wang et al. (2011b), Zhen et al. (2012), Ooms et al. (2013a)
								0.265^b^	
Hap III	Δ	R	R	D	D	No	No	0.070^a^	OhAinle et al. (2008), Wang et al. (2011b), Ooms et al. (2013a)
								0.114^b^	
Hap IV	Δ	L	R	D	D	No	No	0.088^a^	OhAinle et al. (2008), Harari et al. (2009), Wang et al. (2011b)
								0.178^b^	
Hap V	N	R	R	D	E	Yes	Yes	0.202^a^	OhAinle et al. (2008), Wang et al. (2011b)
								0.054^b^	
Hap VI	Δ	L	G	K	D	No	No	0.026^a^	OhAinle et al. (2008), Wang et al. (2011b)
								0.0004^b^	
Hap VII	N	R	R	K	E	Yes	Yes	0.009^a^	OhAinle et al. (2008), Wang et al. (2011b)
								Not detected^b^	

a Wang et al. (2011b).

b OhAinle et al. (2008).

A3H is the only A3 enzyme with highly diversified antiviral activities based on sequence polymorphisms (Li and Emerman, 2011; Duggal et al., 2013) and appears to be in a category of its own in relation to other A3 enzymes regarding two other aspects. First, A3D, A3F, and A3G that also restrict HIV replication have two Z-type domains, whereas A3H has only one Z-domain (LaRue et al., 2008, **Figure 1**). Phylogenic analyses have shown that the A3 Z-type domains have three distinct categories (Z1, Z2, and Z3) and A3H is the only A3 enzyme with an Z3 (LaRue et al., 2008, **Figure 1**). A3D and A3F have two Z2 domains and A3G has an Z1 (CTD) and Z2 (NTD) domain (LaRue et al., 2008, **Figure 1**). Second, A3H is the only single Z-type domain A3 (others are A3A and A3C) that forms oligomers and multimers. Structural and biochemical studies have found that A3A and A3C are largely monomeric (>90%) in solution and do not multimerize in cells through an RNA intermediate (Kitamura et al., 2012; Love et al., 2012; Byeon et al., 2013; Li et al., 2014; Logue et al., 2014; Shlyakhtenko et al., 2014). In contrast, A3H Hap II was found to multimerize in cells (Li et al., 2014). The A3H Hap II multimerization in cells was shown by fluorescence fluctuation spectroscopy and determined that multiple A3H Hap II molecules could closely associate on RNA, not that A3H Hap II oligomerized through a protein–protein interaction (Li et al., 2014). It remains to be determined if A3H Hap II can form a dimer in solution in the absence of RNA or DNA. A3G and A3F form oligomers in the absence of nucleic acid suggesting that A3 oligomerization ability facilitates the multimerization of A3 enzymes with RNA in cells (Chelico et al., 2008; Shlyakhtenko et al., 2011; Ara et al., 2014). It has been shown that similar to A3G and A3F, A3H Hap II interacts with cellular RNA and the NC portion of Gag to facilitate its encapsidation into HIV particles (Wang et al., 2011a; Zhen et al., 2012). Studies on A3H Hap II and Hap I have also shown that cytoplasmic localization correlates with restriction efficiency since mutation of A3H Hap I to make it cytoplasmic (G105R) increases its restriction capacity despite other amino acid differences from A3H Hap II (Harari et al., 2009; Li et al., 2010). Additionally, virion mislocalization of certain A3H haplotypes may render them less active against HIV (Ooms et al., 2010). For example, despite nuclear localization of A3H Hap I, it can be encapsidated into HIV particles, but through an association with the matrix and capsid region of Gag, which leads to its primary localization outside the capsid (Ooms et al., 2010). These data suggest that both cellular and virion localization play a role in restriction efficiency. There has been limited information in the literature on the biochemical properties of A3H and how different haplotypes bind and scan ssDNA in search for deamination targets. A3H Hap II prefers to deaminate ssDNA at 5′TC sites, similar to A3F and A3D, and appears have a high mutagenic potential and ability to restrict HIV in both single-cycle and spreading infection experiments and in HIV-infected individuals (Harari et al., 2009; Hultquist et al., 2011; Wang et al., 2011a; Ooms et al., 2013a).

### RESTRICTION OF HIV BY COORDINATELY EXPRESSED A3 ENZYMES

Vif-deficient HIV showed replication defects when produced from cell lines such as CEM and H9, resulting in their classification as non-permissive cell lines (Gabuzda et al., 1992; Blanc et al., 1993; Sakai et al., 1993; von Schwedler et al., 1993; Madani and Kabat, 1998; Simon et al., 1998). After many years of investigating the function of Vif and trying to understand the dichotomous phenomenon of permissive and non-permissive cell lines for ΔVif HIV, Sheehy et al. (2002) found that the non-permissive CEM cell line expressed A3G. Thereafter, many groups discovered that Vif was required to induce degradation of A3G to enable HIV replication (Sheehy et al., 2002, 2003; Conticello et al., 2003; Kao et al., 2003; Mariani et al., 2003; Marin et al., 2003; Stopak et al., 2003; Yu et al., 2003). Later, it was realized by analyzing the mRNA expression levels of A3s using quantitative PCR in permissive (CEM-SS, SupT1) and non-permissive (CEM and H9) T cell lines that the classical non-permissive CEM T cell line expressed not only A3G but also A3F and A3D, albeit with lower mRNA levels (Refsland et al., 2010). It is interesting to speculate whether more data would be available on the inhibition of HIV by the combined action of multiple A3s if they were discovered at the same time as A3G.

In primary CD4+ T cells A3 enzymes relevant to HIV restriction are expressed and further induced by mitogens, rather than interferon, indicative of their function in restricting retrotransposons (Koning et al., 2009; Refsland et al., 2010). In contrast, in macrophages, monocytes and dendritic cells expression of select A3 enzymes is induced by interferon (Koning et al., 2009; Refsland et al., 2010). Although A3 enzymes are not individually expressed in cells as in many laboratory experiments (Refsland et al., 2010, 2012), there is an advantage of individual expression of each A3. Individual expression of A3 enzymes enables mechanistic information to be learned about enzyme function and mutational footprints established. However, A3s with perhaps a lesser restriction efficiency would not be expressed alone during an HIV infection suggesting that it may not matter *per se* which enzyme is most effective since they may each contribute cooperatively to HIV restriction. Gillick et al. (2013) found that in primary human CD4+ T cells the majority of proviral mutations were in a sequence context that indicated A3G-induced mutations are dominant (5′CC), but A3F- and A3D-induced mutations (5′TC context) were evident at ninefold less frequency than the 5′CC context in ΔVif HIV. This is in contrast to a study by Ooms et al. (2013a) that used peripheral blood mononuclear cells to examine the hypermutation of HIV in the absence or presence of A3H Hap II. In the absence of A3H Hap II, it was found using a deep sequencing approach that there was approximately an equal number of mutations originating in 5′CC and 5′TC contexts, suggesting that A3F and A3D cooperate to induce an equivalent number of mutations to A3G (Ooms et al., 2013a), in agreement with results from a CEM2n T cell line (Refsland et al., 2012). In the presence of Vif that could induce degradation of all A3s except A3H Hap II, there was a large number of mutations in the 5′TC context demonstrating that A3H when present in a stable form is highly active against HIV (Ooms et al., 2013a).

Although the use of spreading infections in primary cells or T cell lines supports the idea that A3s cooperate, there still may be a question of whether they induce HIV evolution. It has been proposed that if there is an insufficient amount of A3-induced hypermutation this may benefit HIV and contribute to sequence variation by induction of sublethal levels of mutagenesis which results in HIV evolution (Mulder et al., 2008; Kim et al., 2010; Sadler et al., 2010; Monajemi et al., 2014). There is evidence that A3G and A3F hotspots are enriched in immunogenic CTL epitopes and that HIV may utilize A3s to induce immune escape (Monajemi et al., 2014). In addition, A3G may be able to induce resistance to the RT inhibitor lamivudine (3TC) because its deamination motif overlaps with a codon for Met and results in an M146I mutation in the *pol* gene (Mulder et al., 2008; Kim et al., 2010). However, the frequency of this mutation being induced by A3G versus RT activity has been questioned (Jern et al., 2009). It is also not known if A3F/A3D/A3H Hap II induce this evolution any more than A3G, due to differences in inactivation potential derived from their sequence specificities (Yu et al., 2004; Zennou and Bieniasz, 2006; Love et al., 2012; Ara et al., 2014) and if this impacts disease progression in infected individuals. On the other hand, Vif has been shown to adapt within HIV-infected individuals and be less effective in inducing A3 degradation (Simon et al., 2005; Fourati et al., 2010). It is thought that HIV can utilize Vif as a mutational rheostat in times of viral stress by allowing low amounts of A3s into viral particles to induce sublethal mutagenesis (Simon et al., 2005; Fourati et al., 2010). These types of studies have raised the idea that perhaps inducing hypomutation or shutting off A3 enzymes may benefit HIV-infected individuals (Harris, 2008; Hultquist and Harris, 2009).

## HIV Vif

### GENERAL PROPERTIES

The main function of Vif remained elusive at the beginning of HIV research, except for the finding that Vif made some cell lines permissive for producing HIV particles capable of undergoing another round of infection (Fisher et al., 1987; Strebel et al., 1987). Non-permissive cells allowed a ΔVif HIV to produce virus particles, but they were rendered non-infectious upon infection of fresh cells. Two laboratories discovered that Vif repressed a host factor (Madani and Kabat, 1998; Simon et al., 1998). It was later identified by subtractive hybridization that A3G (originally called CEM15) was the host factor that was highly packaged into virions in the absence of Vif and blocked infection in the next target cell (Sheehy et al., 2002, **Figure 2A**). Although this is clearly a primary role for HIV infectivity, Vif was also shown to influence HIV particle morphology and this may relate to its potential role as a nucleic acid chaperone (von Schwedler et al., 1993; Hoglund et al., 1994; Henriet et al., 2007; Batisse et al., 2012).

### Vif AS AN E3 UBIQUITIN LIGASE SUBSTRATE RECEPTOR

**FIGURE 5:**
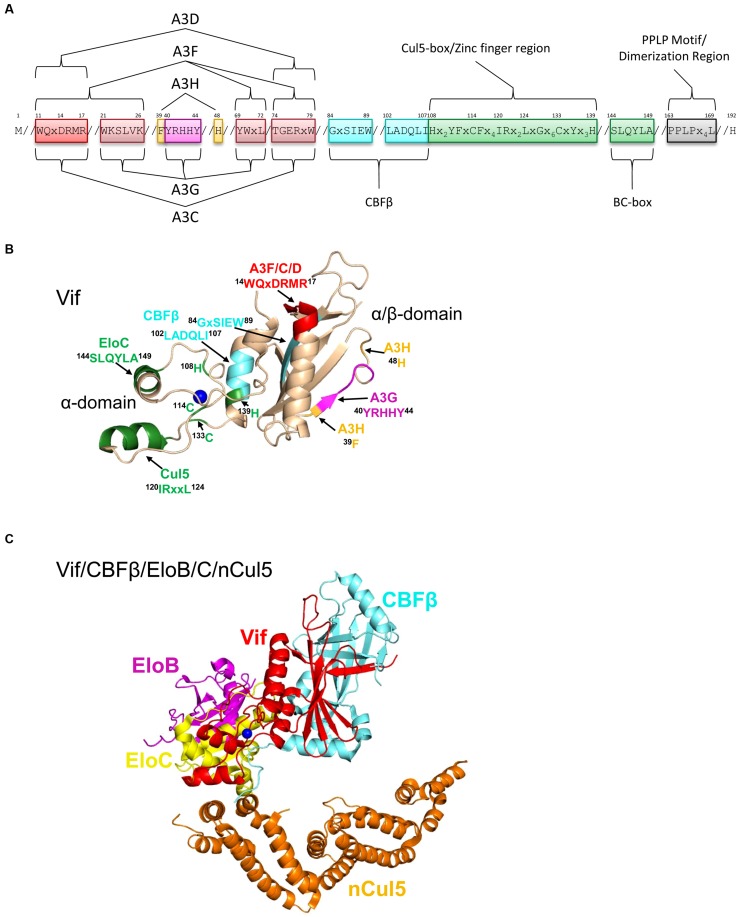
**Structure of Vif and host interacting partners. (A)** Domain organization of Vif. Vif uses specific motifs to interact with A3G (magenta, ^40^YRHHY^44^), A3F/A3C/A3D (red, ^11^WQxDRMR^17^ and ^74^TGERxW^79^), and A3H (orange, ^39^F and ^48^H). In conjunction with these specific motifs, there are shared interaction motifs for A3F and A3G with Vif (pink, ^21^WKSLVK^26^ and ^69^YWxL^72^). CBFβ interacts with Vif through two adjacent motifs (cyan, ^84^GxSIEW^89^ and ^102^LADQLI^107^). The Zinc finger region (green, amino acids 108-139) coordinates the Zinc through an ^108^H^114^C^133^C^139^H motif and stabilizes the Vif structure, which indirectly enables an interaction with Cullin 5 (Cul5). Direct interaction of Vif with Cul5 is through amino acids ^120^IRxxL^124^. The BC box mediates an interaction with Elongin C (green ^144^SLQYLA^149^). Vif oligomerizes through a PPLP motif (gray, ^163^PPLPx_4_L^169^). Slanted lines are used to indicate intervening amino acids between the domains. **(B)** The crystal structure of Vif (PDB: 4N9F) shows that it has two domains on either side of a bound Zinc (blue). The N-terminal α/β-domain consists of a five stranded β-sheet, a discontinuous β-strand and three α-helices. The α/β-domain contains the binding interface for CBFβ (cyan, ^102^LADQLI^107^, ^84^GxSIEW^89^) and A3 enzymes. The ^11^WQxDRMR^17^ motif (red) is used to interact with A3F, A3C, and A3D, the ^40^YRHHY^44^ motif (magenta) is used to interact with A3G, and residues ^39^F and ^48^H (orange) are used to interact with A3H. The α-domain contains two alpha helices that mediate two separate interactions with EloC (green, ^144^SLQYLA^149^) and Cul5 (green, ^120^IRxxL^124^). **(C)** Structure HIV Vif (red) in complex with CBFβ (cyan), Elongin C (EloC, yellow), and the N-terminal domain of Cullin 5 (nCul5, amino acids 12–386, orange, PDB: 4N9F). Elongin B (EloB, magenta) dimerizes with EloC. Figures were made using PyMOL (The PyMOL Molecular Graphics System, Version 1.5.05, Shrödinger, LLC.).

In 2012 it was discovered that Vif interacts with the host transcription cofactor CBFβ for stability in cells (Jager et al., 2012; Zhang et al., 2012). The interaction is mediated through Vif amino acids ^84^GxSIEW^89^ and ^102^LADQLI^107^ (Matsui et al., 2014b; Wang et al., 2014, **Figures 5A,B**). The Vif/CBFβ complex is also required for *in vitro* stability of Vif and enables recombinant expression of Vif in a largely soluble form in *E. coli* that can be purified for biochemical studies (Zhou et al., 2012). In contrast, Vif alone expressed in *E. coli* accumulates in inclusion bodies and must be purified under denaturing conditions (Yang et al., 1996). To suppress A3 action Vif interacts directly with A3 enzymes and mimics the human protein suppressor of cytokine signaling-2 (SOCS2) to become the substrate recognition subunit of a Cullin RING ligase-5 (CRL5) E3 ligase complex (**Figure 2B**).

Vif interacts with host proteins Elongin C, which forms an obligate heterodimer with Elongin B (EloB/C) and Cullin 5 (Cul5, Marin et al., 2003; Yu et al., 2003, 2004; Mehle et al., 2004; Luo et al., 2005; Xiao et al., 2007; Stanley et al., 2008; Bergeron et al., 2010). The interaction of Vif with EloB/C increases the stability of Vif in cells and *in vitro* and promotes recruitment of CBFβ (Wang et al., 2013). The interaction of Vif with EloC is mediated through an SLQ motif in Vif termed the Elongin B/C (BC) box (Yu et al., 2003, 2004, **Figures 5A,B**, ^144^SLQYLA^149^), similar to human SOCS proteins (Kamura et al., 1998; Iwai et al., 1999). Distinct from human proteins is that Vif does not have the highly conserved Cys in the BC box and instead has a ^149^A (Kamura et al., 1998, 2004, **Figures 5A,B**). The data with Vif suggest that it is the short side chain of the amino acid at position 149 rather than the Cys that is required for the interaction with EloC (Yu et al., 2004; Stanley et al., 2008). Vif also does not contain a canonical Cul5 box (Luo et al., 2005; Xiao et al., 2007). In search of the conserved Cys in the BC box, two other Cys (^114^C, ^133^C) were identified in Vif upstream of the BC box and were found to be involved in binding with Cul5 (Mehle et al., 2004; Yu et al., 2004). These Cys were found to be part of a novel Zinc binding HCCH motif (**Figures 5A,B**, ^108^Hx_2_YFxCFx_4_IRx_2_LxGx_6_CxYx_3_H^139^). The Zinc coordination in the HCCH was predicted to stabilize a small domain of Vif and indirectly support Cul5 binding (Luo et al., 2005). The primary Vif amino acids that contact Cul5 are at positions 120–121 and 124 in a helix that is adjacent to the HCCH residues (Xiao et al., 2006; Guo et al., 2014, **Figures 5A,B**, ^120^IRxxL^124^). The Vif/CBFβ/EloB/C heterotetramer undergoes a conformational change that promotes binding to Cul5, suggesting that there is a prescribed order in the assembly of the E3 CRL5 ligase complex (Fribourgh et al., 2014). Accordingly, Cul5 binds less well to EloB/C in the absence of Vif/CBFβ (Guo et al., 2014).

A recent structural study of Vif bound to CBFβ/EloB/C/Cul5 shows that Vif has an overall elongated cone structure and contains two domains with a Zinc binding domain in the center of the two domains (Guo et al., 2014, **Figure 5C**). CBFβ binds the N-terminal α/β-domain and EloC and Cul5 bind the C-terminal α-domain of Vif (Guo et al., 2014, **Figure 5C**). Both EloC and Cul5 interact with Vif through hydrophobic interfaces on distinct α-helices (Xiao et al., 2006; Guo et al., 2014). The crystal structure also emphasizes the stability that CBFβ imparts to Vif since they have a total interaction surface area of 4797 Å^2^ and form an antiparallel β-sheet with a β-strand from each protein (Guo et al., 2014). The side of CBFβ that is bound by Vif is the same side that the human CBFβ binding partner, the RUNX1 transcription factor binds to suggesting a mutually exclusive binding (Kim et al., 2013; Guo et al., 2014), although other reports show CBFβ can bind Vif and RUNX1 on genetically distinct surfaces (Hultquist et al., 2012; Zhang et al., 2012; Du et al., 2013a). Functionally, Vif appears to exclude CBFβ from binding RUNX1 because expression of Vif can alter the RUNX1-dependent transcriptional profile of cells and suggests that Vif may have multimodal effects in HIV-infected cells (Kim et al., 2013).

CBFβ interacts with a hydrophobic region of the Vif α/β-domain, but the rest of the exposed α/β-domain surface is highly positively charged and is thought to mediate electrostatic interactions with A3 enzymes (Aydin et al., 2014; Guo et al., 2014). To target A3s for proteasomal degradation, Cul5 interacts with RING finger protein 2 (Rbx2, Jager et al., 2012) and this results in the assembly of a hexameric complex (**Figure 2B**). Furthermore, an E2 ubiquitin conjugating enzyme interacts with the hexamer through Rbx2 and causes ^48^K-linked polyubiquitination of the A3 enzyme, on multiple lysine residues signaling it for degradation through the proteasome pathway (**Figure 2B**). Current data for A3G and A3F suggest that the Lys residues that become conjugated to ubiquitin are random (Albin et al., 2013).

#### Vif amino acids that interact with A3s

Alanine scanning mutagenesis of Vif or comparison of different Vif variants from HIV subtypes has enabled the identification of three distinct regions of Vif that interact with A3G, A3F/A3D/A3C, or A3H (Huthoff and Malim, 2007; Russell et al., 2009b; Binka et al., 2012, **Figure 5A**). Vif interacts with A3G through two positively charged regions on Vif, ^21^WxSLVK^26^ and ^40^YRHHY^44^ (Mehle et al., 2007; Russell and Pathak, 2007; Yamashita et al., 2008; Chen et al., 2009; Dang et al., 2009, **Figures 5A,B**). Similarly, various domains in Vif have been identified to interact with A3F, specifically ^11^WQxDRMR^17^ and ^74^TGERxW^79^ (Tian et al., 2006; Russell and Pathak, 2007; He et al., 2008; Yamashita et al., 2008, **Figures 5A,B**). In addition, the ^69^YWxL^72^ motif is a region of Vif that interacts with both A3G and A3F (He et al., 2008; Pery et al., 2009, **Figure 5A**). However, for both A3G and A3F mutation of the ^40^YRHHY^44^ and ^14^DRMR^17^ motifs to all alanines are necessary and sufficient to block Vif-induced A3G and A3F degradation, respectively, suggesting the other domains provide a secondary stabilizing interaction (Russell and Pathak, 2007). Of note, A3C and A3D share a common binding site on Vif as A3F with ^14^DRMR^17^ shown to be of importance (Pery et al., 2009; Kitamura et al., 2011, 2012, **Figures 5A,B**). Vif interacts with A3H through another unique site that involves amino acid ^39^F and ^48^H (Binka et al., 2012; Ooms et al., 2013b, **Figures 5A,B**).

The Vif amino acids that interact with A3H are not highly conserved among HIV subtypes, in contrast to the motifs of Vif that interact with A3G and A3F/A3D. It has been suggested that since HIV rarely encounters a host with an active A3H allele, there has been evolutionary drift of Vif to not maintain an interaction site with A3H (Ooms et al., 2013a). As a result, A3H is differentially sensitive to Vif variants. For example, A3H Hap II is not sensitive to HIV NL4-3 Vif (^39^F, ^48^N), but is sensitive to HIV LAI Vif (^39^F, ^48^H, Ooms et al., 2013b). The inability of some Vif variants to induce degradation of A3H Hap II enabled Ooms et al. (2013a) to test whether A3H could act as an infection barrier to HIV. Ooms et al. (2013a) found that Vif will adapt in infected individuals to induce degradation of A3H Hap II (Li et al., 2010). Importantly, this evolution of Vif affects only A3H and Vif maintains the ability to induce degradation of A3G and A3F (Ooms et al., 2013a), confirming that Vif indeed uses three distinct interfaces to interact with A3 enzymes and supports the idea that multiple A3 enzymes coordinately exert a restriction pressure on HIV. Importantly, treatment naïve HIV-infected individuals at the early or primary infection stage that had at least one active A3H allele (Hap II) had higher levels of mutations in proviral genomes in a 5′TC context, lower viral loads and higher CD4+ T cell counts (Ooms et al., 2013a). Gourraud et al. (2011) similarly reported that early stage, untreated HIV-infected individuals that were homozygous for a stable A3H allele demonstrated lower HIV RNA over time, but this did not correlate with increased hypermutation of HIV proviral genomes. This difference in mutational load between these reports is likely due to the different sequencing strategies used between the two studies (Gourraud et al., 2011; Ooms et al., 2013a). These data are similar to clinical data obtained with A3G and A3F that demonstrate in a number of cohorts (but not all), A3G or A3F mRNA expression or hypermutation levels correlate with high CD4+ T cell counts and low viremia (reviewed in Albin and Harris, 2010).

#### Vif–A3G interaction

The Vif–A3G interaction was the first Vif–A3 interaction to be studied and it established that Vif inhibits the antiviral activity of A3 enzymes in a species-specific manner (Bogerd et al., 2004; Mangeat et al., 2004; Schrofelbauer et al., 2004; Xu et al., 2004; Etienne et al., 2013; Letko et al., 2013). This means that HIV Vif cannot neutralize A3G from African green monkey (AGM), and AGM SIV Vif cannot neutralize A3G from humans and this has been recognized as a cross-species infection barrier (Bogerd et al., 2004; Mangeat et al., 2004; Schrofelbauer et al., 2004; Xu et al., 2004; Etienne et al., 2013; Letko et al., 2013). Initially, to identify the residues HIV Vif uses to interact with human A3G the human A3G amino acids were replaced with those of AGM or rhesus macaque A3G. Mutation of human A3G ^128^D to ^128^K as found in AGM and rhesus macaque A3G abrogated the interaction of HIV Vif with human A3G and its ability to induce degradation of human A3G (Bogerd et al., 2004; Mangeat et al., 2004; Schrofelbauer et al., 2004). However, when the ^128^D was mutated to ^128^A, HIV Vif could still interact with and degrade human A3G demonstrating that the charged interface was more important than the amino acid identity (Schrofelbauer et al., 2004). Since mutation of solely ^128^D to ^128^K can abrogate the interaction between A3G and Vif in co-immunoprecipitation studies it is clearly a determining residue. However, Vif-mediated degradation can be influenced by mutation of A3G ^129^P and ^130^D and Vif also interacts with A3G on surrounding motifs such as helix 6 (Huthoff and Malim, 2007; Lavens et al., 2010; Feng et al., 2013, **Figure 3A**). The loop 7 and helix 6 regions contain more positively charged and neutral amino acids than negatively charged amino acids which may explain why ^128^D is such an important contact point for the positively charged Vif, despite a larger A3G interface predicted from biochemical studies. These studies with A3G established the principle that a lack of Vif-induced degradation correlates with a lack of an interaction between the A3 and Vif.

#### Vif–A3F/A3D interaction

A3F and A3D share the same structural motif in the CTD that interacts with Vif (Smith and Pathak, 2010; Kitamura et al., 2012, **Figures 3D,E**). A3F has been studied more extensively than A3D in this regard and will be discussed here. In contrast to A3G, there was no specific single amino acid determinant identified for A3F that clearly mediated both the primary interaction with Vif and was a determinant for Vif-mediated degradation. Rather, different groups identified different amino acids in A3F that altered its susceptibility to Vif. Smith and Pathak (2010) reported that A3F interacts with Vif through CTD amino acids ^289^EFLARH^294^ and that ^289^E was critical for A3F sensitivity to Vif. Albin et al. (2010b) identified another residue, ^324^E, as the key determinant of A3F to Vif-mediated degradation, but mutation of ^324^E to other amino acids, even those of opposite charge, did not disrupt the interaction between A3F and Vif under stringent co-immunoprecipitation conditions. Although other groups have found that the interaction of A3F and Vif could be disrupted at least partially by mutating ^324^E, there was a wider region of A3F that appeared to be important for Vif-mediated degradation in comparison to what was identified for A3G (Albin et al., 2010b; Kitamura et al., 2012; Siu et al., 2013). A combination of mutagenesis, structural modeling and a crystal structure of A3C, that shares the same Vif binding interface with A3F and A3D, identified a novel type of A3 and Vif interaction (Smith and Pathak, 2010; Kitamura et al., 2012). Rather than Vif interacting with a loop as in the case of A3G (**Figure 3A**, loop 7), Vif interacted with a negatively charged surface of A3F/A3D/A3C that spanned helix 2, 3, and 4 and β-strand 4 (Kitamura et al., 2012; Aydin et al., 2014, **Figures 3D,E**). This negatively charged surface supports the hypothesis that it is primarily electrostatic interactions that mediated the A3 and Vif interaction and provides an explanation for why the A3F and Vif interaction may be more difficult to disrupt than the primarily neutral surface present in A3G. It is not known if this would mediate a tighter interaction of Vif with A3F than A3G since there are no quantitative data available for both A3G and A3F using the same experimental conditions (Feng et al., 2013; Siu et al., 2013). Studies with A3F have shown that a lack of Vif-induced degradation does not necessarily correlate with a lack of a Vif-A3F interaction, suggesting that the binding orientation or other factors contribute to successful Vif-mediated degradation rather than only the presence of an interaction (Albin et al., 2010b).

#### Vif–A3H interaction

A3H sensitivity to Vif is haplotype dependent (OhAinle et al., 2008; Harari et al., 2009; Tan et al., 2009; Li et al., 2010; Hultquist et al., 2011; Binka et al., 2012). The A3H Hap I is not sensitive to HIV LAI Vif-mediated degradation whereas A3H Hap II is sensitive to HIV LAI Vif-mediated degradation (Harari et al., 2009; Li et al., 2010; Zhen et al., 2010; Ooms et al., 2013b). The A3H haplotype polymorphisms only occur at three locations (amino acids 105, 121, and 178, **Table 1**). A3H Hap I encodes GKE at these three positions and A3H Hap II encodes RDD at these positions. It was shown that at position 105, the Arg is required for stable expression in cells and that the 178 position had little effect on Vif-mediated degradation (OhAinle et al., 2008; Harari et al., 2009; Li et al., 2010). Therefore, a single amino acid homologous to A3G ^128^D at position 121 in A3H Hap II was determined to control sensitivity to Vif-mediated degradation (Li et al., 2010; Zhen et al., 2010, **Figure 3F**). An A3H Hap II mutant with a ^121^K is not sensitive to Vif-mediated degradation and does not interact with Vif (Zhen et al., 2010). From the A3H structural model (**Figure 3F**), it appears that the ^121^D of A3H is not located on loop 7 as in A3G, but is on helix 4 and on a different face of the molecule (compare **Figures 3A,F**). Yet, similar to A3G the region surrounding ^121^D is mainly neutral or positively charged residues, in contrast to the negatively charged interface that Vif uses to interact with A3F and A3D (Aydin et al., 2014).

### DEGRADATION INDEPENDENT INHIBITION OF A3G

Although Vif primarily inhibits A3G by inducing its proteasomal degradation, there have been other ways in which Vif can inhibit A3G encapsidation or function through a degradation-independent route. Vif may not be able to completely degrade the A3G in the virus-producing cell and these degradation-independent mechanisms may be another line of defense against A3G virion encapsidation. In particular, Vif can become the target of A3-mediated hypermutation (Simon et al., 2005; Jern et al., 2009), which may result in a Vif unable to interact with the E3 CRL5 ligase complex, but still able to inhibit A3G through a degradation-independent mechanism. It is not known if Vif can act in this manner for other A3 enzymes.

#### Vif decreases translation of A3G mRNA

Vif can decrease A3G mRNA translation in order to lower the steady-state levels of A3G through a Vif and A3G mRNA interaction, but the exact mechanism is not understood (Kao et al., 2003; Stopak et al., 2003; Mercenne et al., 2010). It is known that Vif can decrease the mRNA levels of A3G by 15–40% and this requires that Vif interact with the 5′UTR of the A3G mRNA (Stopak et al., 2003; Mercenne et al., 2010). Since Vif has been shown in an immunofluorescence study to co-localize with A3 enzymes and P-bodies (Marin et al., 2008), it is possible that Vif shuttles A3G mRNA to P-bodies to delay or prevent mRNA translation.

#### Vif inhibits virion encapsidation of A3G

Studies by Goila-Gaur et al. (2008) have shown that A3G synthesized *in vitro* using a rabbit reticulocyte lysate translation system would become immunoprecipitation and packaging incompetent in the presence of Vif. Vif was not associated with these high molecular mass A3G forms, but was required for their formation (Goila-Gaur et al., 2008). Although A3G regularly forms high molecular mass complexes in cells, which are less likely to be packaged into virions, Vif can induce an even higher molecular weight form of A3G (Soros et al., 2007; Goila-Gaur et al., 2008). Moreover, studies with an A3G C97A mutant that is resistant to Vif-mediated degradation suggested that Vif-mediated degradation and inhibition of packaging are two distinct properties of A3G since the A3G C97A mutant was encapsidated less well in the presence of Vif (Opi et al., 2007). A molecular mechanism for this effect has not been described.

#### Vif inhibits deamination of deoxycytidine by virion-encapsidated A3G

A3 enzymes are mainly studied with HIVΔ*vif* in order to observe restriction in single-cycle infectivity assays, but in infected individuals A3 enzymes must contend with Vif. Despite multiple mechanisms that Vif uses to block A3G, it has been shown that A3G is encapsidated in the presence of Vif, albeit in lesser amounts (Nowarski et al., 2008). However, per molecule of A3G there is less deamination activity (Britan-Rosich et al., 2011, **Figure 2E**). This decrease in A3G deamination activity even occurs when A3G and Vif are coexpressed in *E. coli* and mutations are detected with a Rifampicin reversion assay or *in vitro* with purified A3G and Vif, demonstrating that other viral components are not required for the inhibition to take place (Santa-Marta et al., 2005; Britan-Rosich et al., 2011; Feng et al., 2013). Enzymatic studies have shown that Vif can cause a decrease in the specific activity of A3G and that this is due to a combination of Vif competitively binding to the ssDNA substrate and Vif binding directly to A3G (Britan-Rosich et al., 2011; Feng et al., 2013). These are separable functions of Vif since ssDNA-binding studies of the Vif–A3G complex in comparison to each of the components binding ssDNA alone support the hypothesis that Vif bound to A3G is unable to bind ssDNA with high affinity (Feng et al., 2013). Another consequence of Vif binding to A3G is that it disrupts how A3G scans ssDNA in search of cytosines to deaminate (Feng et al., 2013). Vif interacts with the A3G NTD on loop 7, which is required for processive jumping movements (Feng and Chelico, 2011, **Figure 3A**). In a study that used two Vif variants to examine the effect of Vif/CBFβ on the deamination activity of A3G it was found that HIV HXB2 Vif inhibited A3G jumping movements, consistent with an interaction of Vif on loop 7 (Feng et al., 2013). In contrast, HIV NL4-3 Vif inhibited A3G sliding, which is mediated by helix 6, providing functional evidence that beyond the key loop 7 contact residues, ^128^DPD^130^, Vif variants can interact with different regions of A3G (Feng et al., 2013). This appears to have no functional consequence for A3G-mediated degradation (Binka et al., 2012), but provides insights on how variable the Vif variants can be in their extended binding sites on A3 enzymes. This may affect strategies that aim to use small molecule inhibitors of the Vif-A3 interaction as an HIV therapy. Importantly, it has been shown that the specific activity of an A3 enzyme is not of primary importance for high levels of deoxycytidine deamination during reverse transcription (Feng et al., 2013; Ara et al., 2014). Rather, the method of efficiently searching for the cytosines, i.e., the enzyme’s processive mechanism appears to be of more importance (Feng et al., 2013; Ara et al., 2014). All together the data suggest that the mechanism by which Vif inhibits A3G deamination activity in virions is by altering the searching mechanism used to find cytosines on ssDNA. Inhibition of A3G deaminase activity by Vif is likely to result in sublethal mutagenesis of HIV and could contribute to the generation of viral quasispecies and HIV evolution (Sadler et al., 2010; Feng et al., 2013).

## DEVELOPMENT OF SMALL-MOLECULE INHIBITORS FOR A3-BASED HIV THERAPEUTICS

The A3-HIV host and pathogen relationship creates the possibility of developing novel therapeutics (Greene et al., 2008; Albin and Harris, 2010) and high-throughput screening approaches for small-molecule inhibitors have uncovered positive results. There are strategies to induce either A3G-mediated viral hypermutation by disrupting the Vif-A3G interaction (Nathans et al., 2008; Cen et al., 2010; Nowotny et al., 2010; Ejima et al., 2011; Ali et al., 2012; Mohammed et al., 2012; Matsui et al., 2014a) or viral hypomutation by blocking A3G catalytic activity (Li et al., 2012; Olson et al., 2013). For the “therapy by hypermutation” strategy, the rationale is to find small-molecule inhibitors that antagonize Vif function and increase the cellular level of A3G available for virus restriction. A few candidate molecules that recover A3G expression levels and enable HIV restriction in the presence of Vif have been discovered (Nathans et al., 2008; Cen et al., 2010; Nowotny et al., 2010; Ejima et al., 2011; Ali et al., 2012; Mohammed et al., 2012; Matsui et al., 2014a), although there are little biochemical data to understand the mechanism of action. For the “therapy by hypomutation” strategy, small-molecule inhibitors have been designed that target a key residue in A3G (C321) that inhibits the catalytic activity of A3G (Li et al., 2012; Olson et al., 2013). It is thought that decreasing the viral quasispecies that may arise due to A3-mediated mutagenesis can assist in immune clearance of the virus and decrease resistance to antivirals (Harris, 2008; Hultquist and Harris, 2009).

Inhibitors targeted to Vif may only be successful if administered in a cocktail to cycle their use and prevent the development of drug resistance, a long-standing therapy regimen for HIV-1 drugs (Greene, 2007). One strategy to avoid selection of resistant Vif variants is to utilize the anti-HIV potential of each A3 enzyme and design inhibitors that bind different regions of Vif, based on the unique interactions that Vif has with A3G, A3F/D, and A3H (**Figure 5A**). However, more study is required to determine if all A3 enzymes function equally well as individual restriction factors otherwise, the strategy may need to involve inhibiting degradation of all A3 enzymes together to enable a strong restriction pressure on HIV. Development of inhibitors that target the A3 enzymes may be a problematic route when considering A3G since Vif interacts with A3G near the amino acid residues needed for virion incorporation, oligomerization, and processivity (Huthoff and Malim, 2007; Huthoff et al., 2009; Feng et al., 2013, **Figure 3A**). As a result, the inhibitor molecule may decrease A3G anti-HIV activity. It is unknown whether the activity of A3D, A3H, or A3F would be affected by this type of strategy. Furthermore, it has been shown in the case of A3G, that HIV can overcome the restriction pressure of A3G by acquiring mutations in genetic sequences other than Vif in order to indirectly avoid A3G encapsidation (Hache et al., 2008, 2009).

If Vif were unable to interact with CRL5 E3 ligase complex components, the accelerated degradation of A3G would be blocked. This strategy has been raised as a potential option (Greene et al., 2008; Bergeron et al., 2010; Zuo et al., 2012) and current structural data on the EloB/C-, Cul5-, and CBFβ- Vif interfaces could facilitate development of inhibitors (Stanley et al., 2008; Guo et al., 2014). However, the consequence of targeting the host proteins with small molecules remains unknown. In addition, this approach has potential drawbacks since Vif may remain bound to the A3 enzymes. For A3G, this has been shown to lead to a decrease in mutagenic activity (Britan-Rosich et al., 2011; Feng et al., 2013). There are no published studies investigating whether Vif would affect the mutagenic activity of other A3s. If Vif were unable to interact with CBFβ it would become unstable in the host cells and degradation of A3 enzymes would be circumvented (Jager et al., 2012; Zhang et al., 2012). However, targeting a small molecule to CBFβ may be problematic if this prevents CBFβ from functioning as the transcription cofactor for RUNX proteins. Although some reports show that Vif and RUNX1 interact with CBFβ on distinct surfaces (Hultquist et al., 2012; Zhang et al., 2012; Du et al., 2013b), Kim et al. (2013) demonstrated that Vif recruitment of CBFβ alters the transcriptional profile of the cell by preventing RUNX1 and CBFβ association.

## PERSPECTIVES

A3 enzymes have the potential to be manipulated as a therapeutic mechanism to suppress HIV replication. Over the past decade, an immense amount of information has been learned regarding each individual A3 enzyme. Cellular, biochemical, and structural data have provided insights on how A3 enzymes interact with nucleic acids and Vif and these data can be strategically applied to develop novel therapies. Critical to predicting the success of an A3-based strategy requires long-term culture of the virus with the potential small molecules to identify tactics HIV could use to overcome the suppression. Another critical facet is understanding if it is necessary for A3 enzymes to work together to restrict HIV *in vivo* in order to invoke the most restrictive pressure on the virus and prevent sublethal mutagenesis.

## Conflict of Interest Statement

The authors declare that the research was conducted in the absence of any commercial or financial relationships that could be construed as a potential conflict of interest.
